# Topical Recombinant Collagen Following Fractional Radio Frequency Microneedling for Nonsurgical Facelift: Case Report

**DOI:** 10.2196/86892

**Published:** 2026-06-29

**Authors:** Thien-Chong Marcus Wong, Yizhi Ong

**Affiliations:** 1 Wong's Plastic Surgery Centre, Gleneagles Hospital, Singapore, Singapore; 2 Department for Continuing Education, University of Oxford, Rewley House, 1 Wellington Square, Oxford, OX1 2IA, United Kingdom, +44 7428612914

**Keywords:** recombinant human collagen, postprocedure recovery, fractional radio frequency microneedling, wound healing, nonsurgical facelift

## Abstract

**Background:**

Fractional radio frequency microneedling (FRM) is an effective nonsurgical facelift modality supported by growing clinical evidence. While generally well tolerated, postprocedural erythema, pain, swelling, and bruising typically last up to 7 days. Other serious complications include prolonged swelling, infection, persistent grid mark, burns, and hypo- or hyperpigmentation. Recombinant human collagen has demonstrated potent wound-healing and anti-inflammatory properties, which could be harnessed for post-FRM recovery.

**Objective:**

To date, there is a lack of published studies evaluating the use of topical agents to improve postprocedural recovery following FRM. This case report describes the early experience of using topical recombinant collagen post FRM for nonsurgical facelift.

**Methods:**

Two 40-year-old female patients with Fitzpatrick skin type IV underwent FRM for nonsurgical facelift. The procedure was performed with standard energy settings. Immediately post procedure, topical recombinant collagen was applied to the treated areas. Either clinical photography or cross-polarized imaging was used to objectively capture the changes in facial erythema and swelling.

**Results:**

Both cases demonstrated a marked reduction of erythema and inflammation within 1 to 2 hours after application of topical recombinant collagen. These observations appeared earlier compared to the typical timeframe of 1 to 3 days. No adverse events were reported.

**Conclusions:**

Topical recombinant collagen application post FRM was associated with an early reduction of skin erythema and inflammation.

## Introduction

In recent years, fractional radio frequency microneedling (FRM) has become a widely recognized and highly effective nonsurgical facelift treatment [1-3]. Studies have shown that it is a useful modality for skin tightening and dermal remodeling without the risks associated with invasive surgery [1-3]. For FRM, controlled thermal energy via microneedles is delivered into the dermis, inducing microinjury to stimulate neocollagenesis and elastin production [1-3]. Although its clinical efficacy and tolerability have been well documented, postprocedural erythema, edema, bruising, and discomfort are common and may persist for up to several days [4]. Other rare but serious complications that have been observed include prolonged swelling, persistent grid marks, burns, infection, and hypo- or hyperpigmentation [4].

Currently, no standardized postprocedural care protocol has been established to accelerate recovery and reduce downtime [4], underscoring the unmet clinical need for biologically active adjuncts that support tissue repair and reduce inflammation. Recombinant human collagen is an emerging innovative biomaterial that has shown promising potential for improving wound healing while minimizing inflammation [5-8], which could be harnessed for post-FRM recovery following energy-based facial rejuvenation procedures. To our knowledge, the use of topical recombinant collagen in the immediate post-FRM setting has not been described in the medical literature. Herein, we describe an innovative postprocedure approach using topical recombinant collagen in two patients who underwent a nonsurgical facelift with FRM.

## Case Presentation

### Overview

**Figure 1.**
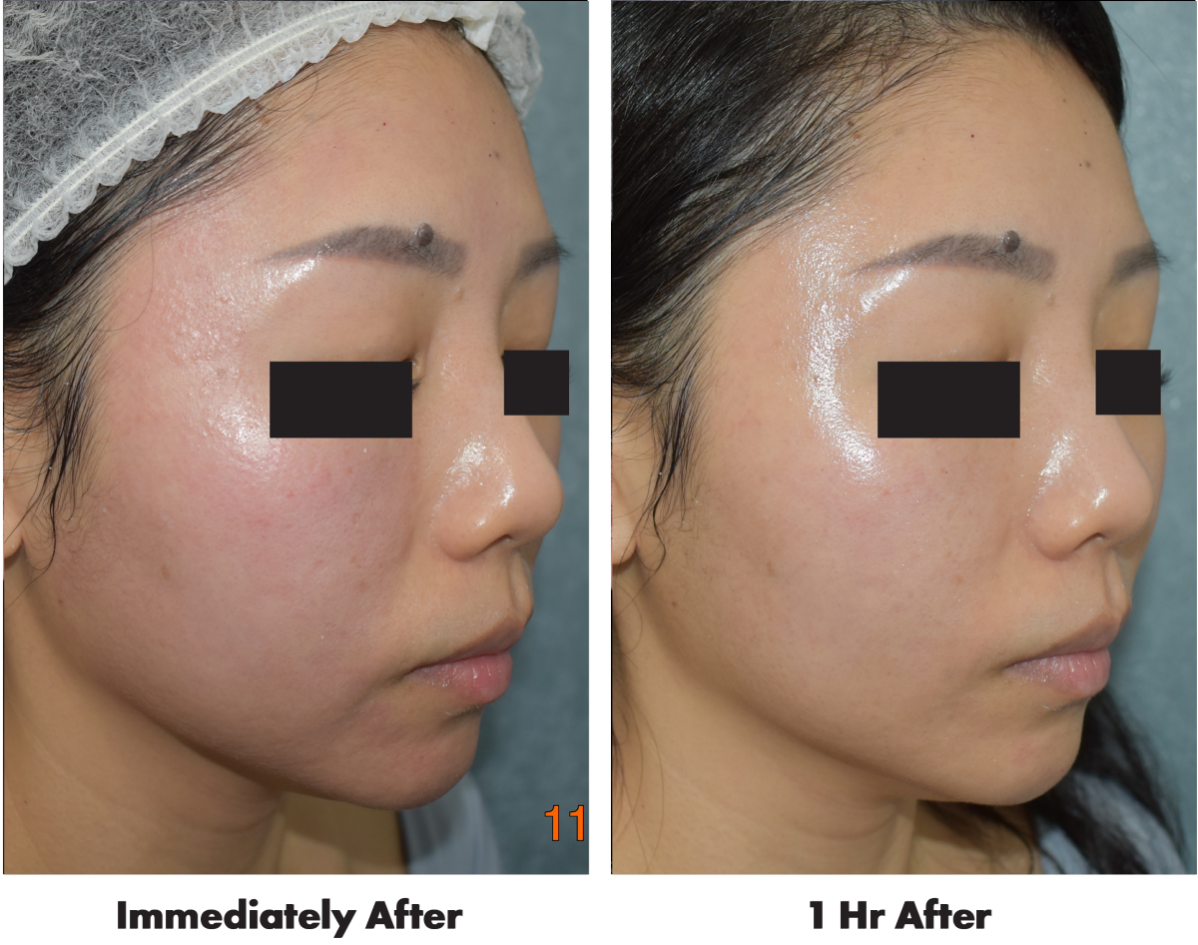
Clinical photography under standard lighting capturing apparent reduction in facial erythema and edema within 1-hour post fractional radio frequency microneedling.

**Figure 2.**
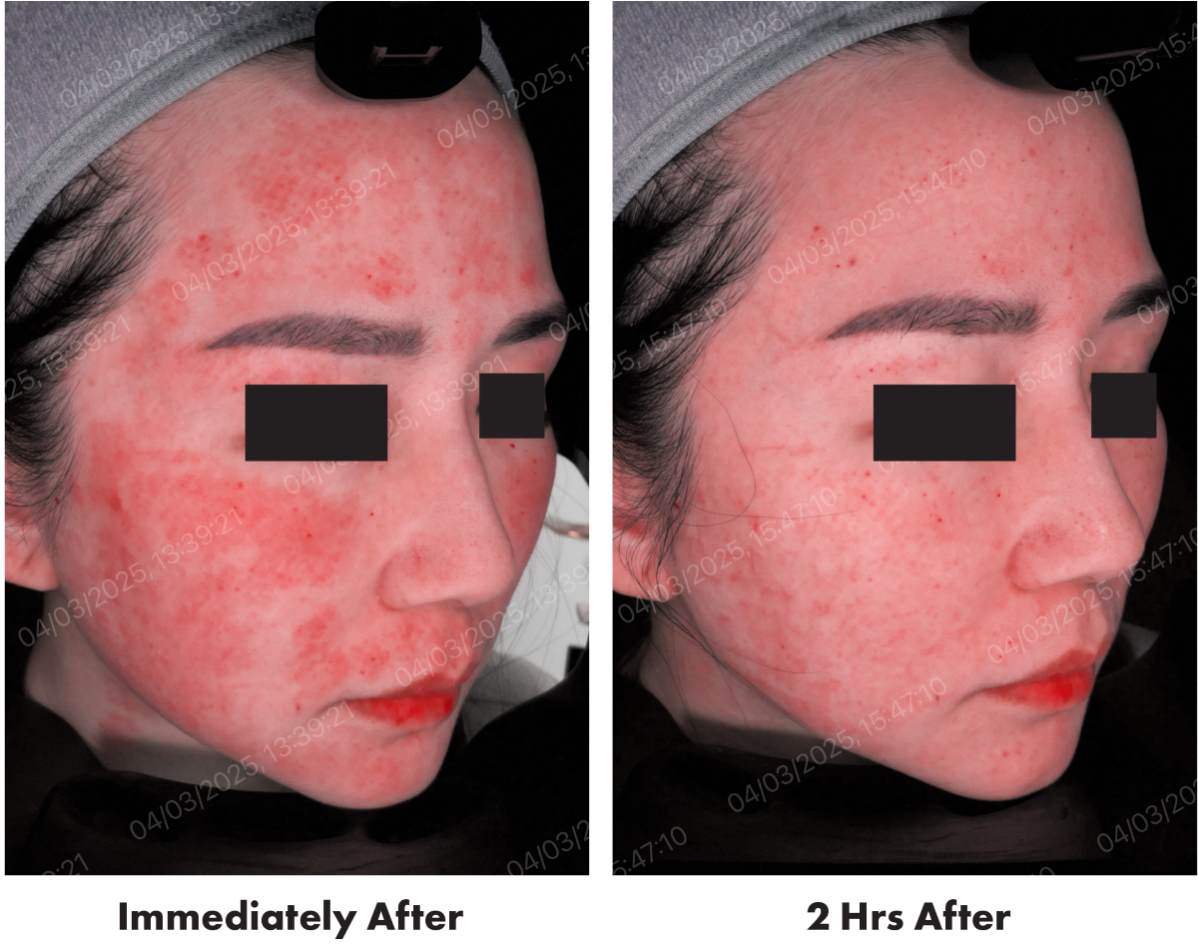
Marked improvement of skin inflammation visualized by a 3D skin analyzer under cross-polarized light within 2 hours post fractional radio frequency microneedling.

Two 40-year-old female patients with Fitzpatrick skin type IV presented with facial laxity and underwent FRM for nonsurgical facelifting. Both were nonsmokers, with no significant past medical history or drug allergies. The baseline assessment documented mild-to-moderate facial laxity with fine lines around various parts of the face. Standardized clinical photography under consistent lighting or cross-polarized imaging was performed before treatment, immediately after treatment, and 1 to 2 hours after treatment. The procedure was performed using a 24-pin tip in a fixed mode, with two passes and 35% to 50% overlap. They received a total of approximately 600 pulses each with standard energy settings of 20 to 25 mJ at a 2.0 mm depth for facial bony areas and 30 to 45 mJ at a 3.0 to 4.0 mm depth for facial soft tissue regions. Immediately post procedure, topical recombinant collagen was applied to both patients as part of their routine posttreatment care. The multimodal documentation showed early observable changes in erythema and inflammation within 1 to 2 hours after application of topical recombinant collagen. Case 1 demonstrated a clinically significant reduction in facial edema and erythema (Figure 1) that was observed on clinical photography within 1 hour post treatment. Additionally, the patient reported immediate soothing of skin discomfort and burning sensation after application of topical recombinant collagen. Case 2 showed a marked reduction of inflammation (Figure 2), visualized using a 3D skin analyzer under cross-polarized light within 2 hours post treatment. Subjectively, the patient reported rapid relief of skin redness and irritation after application of topical recombinant collagen. No adverse events or side effects were reported. The procedure and posttreatment care were well tolerated by both patients. During the follow-up review, both patients were satisfied with the treatment and rapid recovery results. The CARE Checklist has been completed for the drafting of this case report.

### Ethical Considerations

This case report did not require institutional ethics review according to the National Healthcare Group Domain Specific Review Board guidelines, as it describes observations from routine clinical care without systematic research intervention.

Written informed consent was obtained from the patients for publication of this case report and any accompanying images.

## Discussion

This novel case report has shown that topical recombinant collagen applied immediately post FRM was associated with clinically apparent reduction in erythema, edema, and discomfort within 1 to 2 hours post treatment as compared to the expected 1 to 3 days [1-3]. The temporal relationship between the application of topical recombinant collagen and the earlier-than-expected postprocedural reduction in erythema and inflammation could represent a plausible effect superimposed on the normal recovery process. The reduction in skin erythema was apparent to the attending clinician, which was notably different from the typical recovery trajectory of FRM. The observed objective and subjective improvements suggest that topical recombinant collagen may be a feasible adjunct for postprocedural care following FRM. Collagen-based biomaterials have been shown to promote wound healing, stabilize the extracellular matrix, and reduce inflammation in both acute and chronic wounds [5-7]. While previous studies have largely focused on burns or surgical wounds [5-7], our findings have extended the potential application of recombinant collagen to aesthetic procedures involving controlled dermal injury from FRM. This early clinical experience corroborated with known in-vitro dermal repair and anti-inflammatory mechanisms of recombinant collagen, including the modulation of inflammatory cytokines such as inducible nitric oxide synthase, interleukin 1β (IL-1β), IL-10, and tumor necrosis factor α [9]. Postprocedural downtime is an important consideration. These preliminary observations warrant further investigation into strategies to support recovery following FRM.

Nevertheless, this report has several limitations. Only two patients were included with short-term follow-up. There was no control group for comparison. No standardized scoring systems for erythema, swelling, or patient-reported outcomes were used, as this was not part of routine clinical documentation. Randomized controlled studies will be required to confirm treatment safety and efficacy while optimizing treatment protocols before routine implementation.

In summary, topical recombinant collagen may represent a potential adjunct in postprocedural care following FRM, warranting further evaluation in prospective clinical trials.

## Abbreviations

FRM: fractional radio frequency microneedling
IL-1β: interleukin-1β
